# Oxytocin modulation of resting-state functional connectivity network topology in individuals with higher autistic traits

**DOI:** 10.1093/psyrad/kkaf021

**Published:** 2025-08-08

**Authors:** Abraham Tonny Hagan, Lei Xu, Juan Kou, Yuan Hu, Benjamin Klugah-Brown, Jialin Li, Mercy Chepngetich Bore, Benjamin Becker, Keith M Kendrick, Xi Jiang

**Affiliations:** The Clinical Hospital of Chengdu Brain Science Institute, MOE Key Laboratory for Neuroinformation, School of Life Science and Technology, University of Electronic Science and Technology of China, Chengdu 611731, China; The Clinical Hospital of Chengdu Brain Science Institute, MOE Key Laboratory for Neuroinformation, School of Life Science and Technology, University of Electronic Science and Technology of China, Chengdu 611731, China; Institute of Brain and Psychological Sciences, Sichuan Normal University, Chengdu 610066, China; Institute of Brain and Psychological Sciences, Sichuan Normal University, Chengdu 610066, China; The Clinical Hospital of Chengdu Brain Science Institute, MOE Key Laboratory for Neuroinformation, School of Life Science and Technology, University of Electronic Science and Technology of China, Chengdu 611731, China; The Clinical Hospital of Chengdu Brain Science Institute, MOE Key Laboratory for Neuroinformation, School of Life Science and Technology, University of Electronic Science and Technology of China, Chengdu 611731, China; The Clinical Hospital of Chengdu Brain Science Institute, MOE Key Laboratory for Neuroinformation, School of Life Science and Technology, University of Electronic Science and Technology of China, Chengdu 611731, China; Department of Psychology, the University of Hong Kong, Hong Kong, China; State Key Laboratory of Brain and Cognitive Sciences, The University of Hong Kong, Hong Kong, China; The Clinical Hospital of Chengdu Brain Science Institute, MOE Key Laboratory for Neuroinformation, School of Life Science and Technology, University of Electronic Science and Technology of China, Chengdu 611731, China; The Clinical Hospital of Chengdu Brain Science Institute, MOE Key Laboratory for Neuroinformation, School of Life Science and Technology, University of Electronic Science and Technology of China, Chengdu 611731, China

**Keywords:** autistic traits, oxytocin, resting-state fMRI, graph theory, topology measures

## Abstract

**Background:**

Altered connectivity patterns in socio-emotional brain networks are characteristic of individuals with autism spectrum disorder. Despite recent research on intranasal oxytocin's modulation effects of network topology in autism, its specific effects on the functional connectivity network topology remain underexplored.

**Methods:**

To address this gap, we conducted an exploratory data-driven study employing a dimensional approach using data from a large cohort of 250 neurotypical adult male subjects with either high or low autistic traits and who had administered 24 IU of intranasal oxytocin or placebo in a randomized, controlled, double-blind design. Resting-state functional connectivity data were analyzed using network-based statistical methods and graph theoretical approaches.

**Results:**

The findings from treatment × autistic trait group interactions revealed significantly different effects of oxytocin in local (cluster coefficient, efficiency, nodal path length, degree and betweenness centrality) but not global graph metrics in individuals with higher autistic traits compared to those with lower ones, across multiple brain regions. Changes across multiple measures were found in the motor, auditory/language, visual, default mode and socio-emotional processing networks, all of which are influenced in autism spectrum disorder.

**Conclusion:**

Overall, findings from this dimensional approach demonstrate that oxytocin particularly targets widespread enhancement of local but not global neural network processing parameters in neurotypical individuals with higher autistic traits. This suggests that intranasal oxytocin may represent a therapeutic option for social, emotional and sensorimotor symptoms in individuals with autism spectrum disorder by modulating local integration within brain regions involved in their regulation.

## Introduction

Autism spectrum disorder (ASD) is a complex neurodevelopmental condition characterized by problems in social communication and interaction as well as restricted and repetitive behaviors and interests (Hyman *et al*., 2020). The rising prevalence of ASD (Fombonne, 2018) underscores an urgent need to elucidate its neurobiological underpinnings. Despite extensive research, the etiology of ASD remains elusive due to its heterogeneous symptomatology (Hyman *et al*., 2020). Dimensional measures of autistic traits reveal a continuum of autistic-like behaviors within the general population (Lord *et al*., 2012; Abu-Akel *et al*., 2019), highlighting a critical gap in understanding how brain network functioning varies across these traits. Autistic traits are consistently linked to alterations in brain structure and function, particularly within networks associated with social cognition. Key networks include the default mode network (DMN) (Padmanabhan *et al*., 2017), salience network (Haghighat *et al*., 2021), and theory of mind network (Paul *et al*., 2021). These alterations contribute to problems in social perception, cognition, and behavior (Thaler *et al*., 2020). Moreover, reduced gaze towards social stimuli (Hedger and Chakrabarti, 2021) and altered emotional processing (Del Bianco *et al*., 2021) emphasize the need to unravel the neural mechanisms underlying social cognition in ASD. The absence of established pharmacological interventions for ASD highlights a significant unmet clinical need, making it crucial to explore promising therapeutic approaches and investigate differences in large-scale brain network interactions and topological organization implicated in ASD.

Functional connectivity analysis provides valuable insights into the temporal correlation between brain regions and the intrinsic organization of neural networks (Anzellotti *et al*., 2018; Park and Friston, 2013). Evidence reveals both brain-wide and regional functional connectivity alterations in ASD, affecting short- and long-range connections across various imaging modalities (Jiang *et al*., 2023; Nielsen *et al*., 2013; Zhang *et al*., 2023). For instance, dynamic over- and under-functional connectivity has been identified in key brain regions, such as the postcentral gyrus, insula, cerebellum, caudate nucleus, and temporal pole (Liu *et al*., 2024). Similarly, another study (Li *et al*., 2021) reported altered developmental trajectories associated with cascading neurobiological processes across brain networks, while another highlighted hyperconnectivity involving the amygdala and thalamus (Kaur and Kaur, 2023) in individuals with ASD. Despite these findings, inconsistencies persist, including atypical intrinsic connectivity within the DMN and variable connectivity patterns (Duan *et al*., 2017; Duan and Chen, 2022; Hong *et al*., 2019). Decreased effective connectivity has also been reported among large-scale brain networks (Wei *et al*., 2022), and evolving disconnectivity patterns (Nomi and Uddin, 2015) across the lifespan. While there has been progress towards establishing brain imaging findings which can potentially be used in a diagnostic context (Jiang *et al*., 2023; Schielen *et al*., 2024), these are yet to achieve sufficient accuracy and consistency, which underscores the need for robust statistical approaches, such as network-based statistics, to comprehensively capture neural changes which contribute to symptoms in ASD.

Graph theory has emerged as a powerful tool for studying functional brain network alterations (Bullmore and Sporns, 2009; Sporns and Betzel, 2016). By representing brain regions as nodes and their connections as edges, graph theory quantifies network properties such as global and local efficiency, clustering coefficient, path length, modularity, centrality, and small-worldness (Bassett and Bullmore, 2017). Applying graph theoretical measures to resting-state functional magentic resonance imaging (rs-fMRI) data provides insights into altered functional brain organization in ASD, from macro to micro scales (Canario *et al*., 2021; Di Martino *et al*., 2014; Larivière *et al*., 2019). Typically, the brain's topology comprises a complex network of neurons that communicate through synapses, forming a system crucial for effective information processing (Fornito *et al*., 2016). Evidence has revealed altered network topology in the brain of autistic individuals, affecting robustness in information processing and retrieval (Heiney *et al*., 2021). For instance, reductions in modularity and local efficiency, coupled with higher global efficiency in ASD (Rudie *et al*., 2012) and altered small-world regimes and nodal centralities within the DMN (Chen *et al*., 2021) have been reported. These findings suggest that ASD involves both global and local functional topological network differences, highlighting the need for interventions targeting both.

Intranasal oxytocin (OT) has emerged as a potential therapeutic agent due to its role in social behavior and emotional regulation (Kendrick *et al*., 2018; Yao and Kendrick, 2025). Produced in the hypothalamus and released into the bloodstream and brain, OT is important for social bonding, cognition, and emotional processing (Jurek and Neumann, 2018; Kendrick *et al*., 2018; Yao and Kendrick, 2025). OT receptors are densely distributed in brain regions involved in social cognition, such as the medial prefrontal cortex, amygdala, insula, hippocampus, cingulate cortex, and basal ganglia (Gimpl and Fahrenholz, 2001; Jurek and Neumann, 2018; Quintana *et al*., 2019). Studies have reported that intranasal OT facilitates a number of social behaviors, emotion recognition, and attention towards salient social stimuli (see Yao and Kendrick, 2025). Additionally, OT may have anxiolytic and social buffering effects (Naja and Aoun, 2017; Riem *et al*., 2020) and improve social and emotional functioning in ASD.

Notably, some clinical trials have reported improvements in social symptoms in individuals with ASD following chronic intranasal OT treatment, including increased visual attention to social stimuli (Le *et al*., 2022; Parker *et al*., 2017; Yamasue *et al*., 2020; Yatawara *et al*., 2016). However, despite overall evidence for improved social symptoms, findings have been inconsistent (Audansdottir *et al*., 2024). OT has also been shown to facilitate the pleasantness of social touch and corresponding brain reward responses which are also often atypical in individuals with ASD (Chen *et al*., 2023; Thye *et al*., 2018). Moreover, intranasal OT has been shown to affect amygdalo-hippocampal functional connectivity (FC) in both neurotypical and autistic individuals (Alaerts *et al*., 2019; Coenjaerts *et al*., 2023). Overall, these studies suggest that OT administration can potentially improve social symptoms by modulating functional connectivity within key brain regions involved in social and emotional processing and could therefore produce therapeutic benefits in individuals with ASD. However, the full impact of OT on brain network topology remains underexplored.

Autistic symptoms vary across both non-clinical and clinical populations (Constantino and Todd, 2003; Lundström *et al*., 2012) and therefore a dimensional approach can be used to learn about neural contributions to clinical autism by studying the impact of varying severities of autistic traits in neurotypical individuals. Autistic traits can be assessed using the Autism Spectrum Quotient (ASQ) (Baron-Cohen *et al*., 2001) and higher ASQ scores are associated with greater social challenges, rigid behaviors, and heightened sensory sensitivity, whereas lower scores correlate with greater behavioral flexibility and reduced sensory sensitivity. Individuals with higher autistic traits may exhibit compensatory mechanisms, such as enhanced mirror neuron system (MNS) responses, which could potentially counterbalance amygdala dysfunction often observed in individuals with ASD (Fernández *et al*., 2018; Xu *et al*., 2022; Zhao *et al*., 2023). The MNS, which is involved in understanding and imitating the actions of others, may be more active in individuals with higher autistic traits, compensating for social processing deficits associated with amygdala dysfunction. The amygdala, crucial for emotional and social processing, often shows altered functioning in individuals with ASD, contributing to challenges in interpreting social cues (Fernández *et al*., 2018). Given that the amygdala is a primary target for OT, it is plausible that OT could modulate network topology in individuals with autistic traits by enhancing connectivity between the MNS and the amygdala, potentially improving social and emotional processing. Research suggests individuals with ASD may have lower OT concentrations compared to neurotypical individuals (Moerkerke *et al*., 2021). Additionally, variations in the oxytocin receptor (OXTR) gene could impact OT binding and signaling, potentially influencing social and emotional functions by altering neural activity and connectivity in various brain regions (Jurek and Neumann, 2018).

Despite substantial evidence from diverse methodologies, effective therapeutic interventions for individuals with ASD that can influence neural contributions to the disorder have yet to be established. Small-scale graph theoretical studies suggest OT may modulate regional processing within relevant cognitive, emotional, attentional, theory of mind, and sensory networks (Martins *et al*., 2021; Zheng *et al*., 2021). However, studies to date have often involved limited cohorts, or focused on specific brain regions, or have not assessed the potential influence of autistic traits, underscoring the need for more comprehensive investigations in larger groups with identified levels of sub-clinical autistic traits. Specifically, one study focused exclusively on frontal regions (Zheng *et al*., 2021), while another (Martins *et al*., 2021) conducted a whole-brain study but with a small sample size, limiting the generalizability of their conclusions. Both studies highlighted that OT generally increased integration between key networks such as the fronto-parietal network (FPN), default mode network (DMN), sensorimotor network (SMN), and limbic system. In a previous study (Hagan *et al*., 2025) in a large cohort of neurotypical adult male subjects we have also reported that intranasal OT modulates inter-regional functional patterns, small-worldness, and regional organization of topographical networks in the resting-state human brain. The modulatory effects of OT were associated with widespread brain regions, in frontal, parietal, and occipital cortices as well as sub-cortical and cerebellar regions. However, this initial study did not consider the influence of individual autistic traits. Thus, to date it remains unexplored whether intranasal OT may particularly influence neural network processing in individuals with higher autistic traits.

To address this gap, our exploratory and data-driven study employed graph theoretical techniques on rs-fMRI data in a large cohort of subjects to investigate the whole-brain effects of intranasal OT on FC and network topology in individuals with higher and lower autistic traits, as measured by the widely used ASQ. We hypothesized that OT would particularly modulate local but not global integration measures in individuals with higher autistic traits, as measured by the ASQ.

## Materials and methodology

### Participants

Data from 250 non-smoking, healthy adult male participants were included who satisfied the criteria for inclusion as having either higher or lower autistic traits. Resting state data included in the current study were taken from the 139 adult males in one previous study (Hagan *et al*., 2025) and from a further 111 from another study in the laboratory (Kou *et al*., 2022) scanned during the same period using the same MRI scanner and resting-state scanning parameters (Kou *et al*., 2022). All included participants were originally recruited through advertisement at the University of Electronic Science and Technology of China. Only males were included to align with most other OT resting-state studies and to avoid potential confounding factors related to the menstrual cycle in females. All participants were right-handed and self-reported having no current or previous mental health problems, neurological disorders, or other medical conditions. Participants were further screened for neurological or psychiatric conditions during face-to-face interviews with a psychological counselor prior to the study. Individuals with any history of such conditions were excluded. To control for confounding factors, all participants were free from MRI contraindications and were instructed to avoid alcohol, caffeine, nicotine, and other psychoactive substances for at least 24 h before the experiment.

The two studies contributing to the data for the subjects included in the current study were approved by the ethics committee at the University of Electronic Science and Technology of China, and all participants provided written informed consent. The study protocols all adhered to the latest revision of the *Declaration of Helsinki*.

### Study design and data sources

This exploratory, data-driven study utilized rs-fMRI data from two pre-registered clinical trials (NCT02741063 and NCT03610919), which originally investigated the effects of intranasal OT on attentional performance and face emotion recognition. While the original trials did not preregister hypotheses concerning the effects of OT on resting-state brain connectivity on individuals with different autistic traits, rs-fMRI data were systematically collected prior to task performance in each study using standardized acquisition protocols across all participants. The current analysis includes all eligible participants from these original trials, which used identical scanning parameters. This integrative approach enabled us to examine a key, underexplored question: how does OT modulate large-scale brain connectivity across individual differences in autistic traits within a large non-clinical population? Both studies employed a randomized, double-blind, placebo-controlled (OT–PLC) design and participants were randomly assigned to receive a single dose of intranasal OT (24 IU) or placebo. Each participant completed questionnaires during an initial visit, followed by fMRI scanning during a second visit. This protocol ensured rigorous control over treatment administration and minimized potential sources of bias.

Post-hoc stratification based on autistic trait levels was conducted independently of treatment assignment, maintaining the integrity of the original randomization. By adopting a dimensional (rather than categorical) approach, we aimed to explore the modulatory effects of OT in individuals with high as opposed to low autistic traits. This strategy provides a more nuanced understanding of the impact of OT on functional brain networks in a non-clinical population.

### Autistic trait stratification

All participants completed the ASQ prior to treatment and scanning and were stratified into higher and lower autistic trait groups based on their scores. Higher ASQ scores reflect greater autistic traits, with scores above 32 typically considered indicative of potential clinical-level symptoms (Sheldrick *et al*., 2020; Thabtah *et al*., 2019). To capture the natural variability of autistic traits within a non-clinical population, we applied a post-hoc stratification approach by selecting participants in the highest and lowest 30% of the ASQ score distribution from over 400 individuals across the two prior studies. From the 250 participants who completed all required sessions (out of >400 ASQ respondents), 109 individuals with ASQ scores ≥25 were assigned to the higher trait group, and 138 individuals with scores ≤18 to the lower trait group. Stratification was conducted independently of treatment allocation, preserving the randomized, double-blind, placebo-controlled design.

### Treatment allocation and psychological assessments

Within each trait group, participants had been randomly assigned to receive intranasal OT (24 IU) or placebo (PLC) according to standardized protocols (Guastella *et al*., 2013). OT and PLC sprays were provided by Sichuan Defeng Pharmaceutical Company (Chengdu, Sichuan, China). In total, 62 participants in the higher trait group and 94 participants in the lower trait group received OT, with the remainder receiving PLC.

Additional psychological questionnaires were administered prior to treatment and scanning to characterize mood, anxiety, and empathy profiles across groups. These included the Beck Depression Inventory-II (BDI-II) (Beck *et al*., 1996), State-Trait Anxiety Inventory (STAI) (Spielberger, 1983), Positive and Negative Affect Schedule (PANAS) (Watson *et al*., 1988), and the Empathy Quotient (EQ) (Allison *et al*., 2011).

### fMRI data acquisition

rs-fMRI scanning commenced 45 min following nasal spray administration, aligning with the time point at which OT is believed to reach peak central concentrations. This timing is consistent with existing recommendations (Guastella *et al*., 2013) and has been widely adopted in previous research (Quintana *et al*., 2019; Riem *et al*., 2020; Jiang *et al*., 2021; Xin *et al*., 2021; Hagan *et al*., 2025). During the scan, participants were instructed to remain relaxed with their eyes open, while avoiding sleep or deliberate thought. Imaging data were acquired using a 3T GE MR750 Discovery MRI system (General Electric Medical Systems, Milwaukee, WI, USA). Functional images were collected with a gradient-echo planar imaging (EPI) sequence during the resting-state paradigm [38 interleaved slices; repetition time (TR) = 2 s; echo time (TE) = 30 ms; field of view (FOV) = 252 × 252 × 133 mm; matrix size = 80 × 80 × 38; flip angle = 90°; in-plane resolution = 3.15 × 3.15 mm; slice thickness = 3.5 mm; no inter-slice gap]. The resting-state scan lasted approximately 8 min, yielding a total of 240 time points. Additionally, whole-brain high-resolution three-dimensional T1-weighted anatomical images were obtained using an MP-RAGE sequence (sagittal acquisition; FOV = 240 × 240 × 170 mm; voxel size = 1 × 1 × 1 mm, isotropic). Image processing and statistical analysis were conducted using Statistical Parametric Mapping software (SPM8; www.fil.ion.ucl.ac.uk/spm).

### fMRI data preprocessing and functional connectivity analysis

Standard preprocessing procedures were applied using the Functional Connectivity Toolbox (CONN; http://www.nitrc.org/projects/conn/; Whitfield-Gabrieli & Nieto-Castanon, 2012). These included slice-timing correction, motion correction with artifact rejection (using a scan-to-scan motion threshold of 2 mm), spatial normalization, and spatial smoothing with an 8 mm full-width at half-maximum (FWHM) Gaussian kernel. Prior to statistical analysis, data were denoised using the anatomical component-based noise correction method (aCompCor), which accounts for physiological and other non-neuronal noise by regressing out components derived from white matter and cerebrospinal fluid signals (Behzadi *et al*., 2007).

Data from all 247 participants were included in the analysis. Brain signals were extracted from 116 regions of interest defined by the Automated Anatomical Labeling (AAL) atlas. To assess the effects of OT on intrinsic functional connectivity, Pearson correlation coefficients were calculated between the mean fMRI time series of each pair of the 116 regions. These correlation values were then transformed to z-scores using Fisher's r-to-z transformation to ensure normality. The resulting Z-matrices exhibited Gaussian distributions, and the data were subsequently screened for outliers prior to statistical testing.

To identify differences in whole-brain functional connectivity, a network-based statistics (NBS) approach was employed. This analysis compared participants with higher versus lower autistic traits who received OT versus PLC, with age and head motion quantified as mean framewise displacement (FD) included as covariates in an analysis of covariance (ANCOVA) model. Statistical significance was assessed at a family-wise error (FWE) corrected threshold of *P* < 0.05.

### Graph construction and functional brain network analysis

The z-transformed functional connectivity matrices were used to construct individual brain graphs, representing each participant's functional connectome. From these matrices, network properties were derived for each subject. To generate undirected adjacency matrices, the functional connectivity metrics were binarized: connections with correlation values exceeding a defined proportional threshold were assigned a value of 1, and those below were set to 0. This binarization process served to retain only the strongest functional connections within the network.

To ensure robust and unbiased estimation of network topology, a range of sparsity thresholds—from 10% to 50%, in 1% increments was applied to the correlation matrices. This approach reduces the likelihood of spurious edges and facilitates reliable calculation of small-world network parameters (Feng *et al*., 2012). Sparsity was defined as the proportion of existing edges relative to all possible connections in the network. For each treatment group, a mean connectivity matrix was generated. Subsequently, both global and nodal network metrics were computed at each sparsity level. Global metrics included clustering coefficient, characteristic path length, global efficiency, and local efficiency. Nodal-level metrics included nodal efficiency, nodal degree, and betweenness centrality. However, specific graph-theoretical measures were selected for hypothesis testing: clustering coefficient, characteristic path length, nodal efficiency (both local and global), nodal degree, and betweenness centrality.

**Figure 1:**
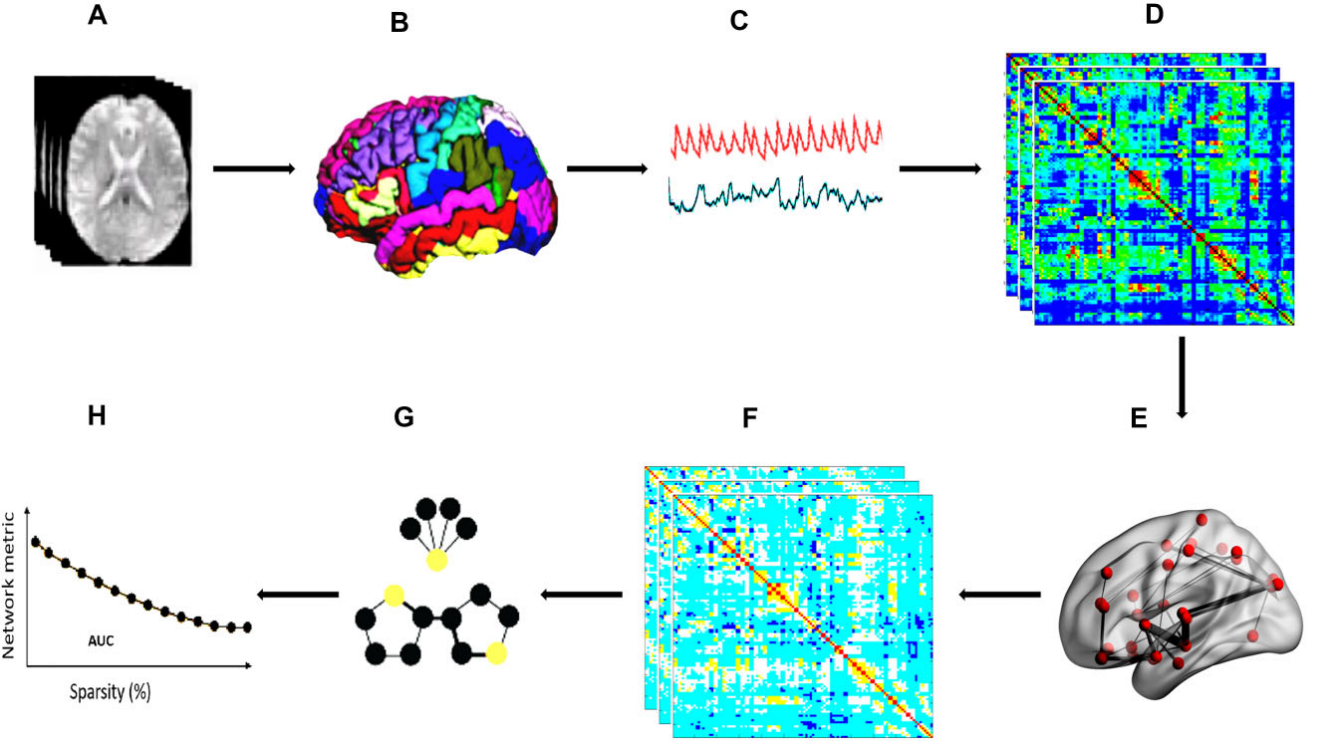
Workflow for network topology analysis. (**A**) rs-fMRI; (**B**) parcellation; (**C**) time series extraction; (**D**) connectivity metrics; (**E**) brain graph calculation; (**F**) functional connectivity metrics thresholding; (**G**) topology network metrics; (**H**) area under the curve (AUC) of metrics.

All graph computations were performed using the GraphVar toolbox (Kruschwitz *et al*., 2015). To enable a more detailed and hypothesis-driven interpretation, network metrics were reported separately for each sparsity threshold, along with their corresponding correlation coefficients (*r*-values) and significance levels (*P*-values). This approach allows for a comprehensive evaluation of functional connectivity patterns by capturing a spectrum of network strengths—ranging from strong, well-defined connections to subtler, potentially meaningful effects. It also provides a clearer indication of the specific sparsity levels at which OT exerts its most pronounced influence on brain network architecture. A visual summary of the full analytical workflow is presented in Fig. 1.

### Statistical analysis

The study employed a comprehensive statistical approach that integrated NBS, two-sample t-tests, and permutation testing to assess treatment effects and interactions between trait groups, while carefully controlling for potential confounding factors associated with common comorbidities in individuals with autism, such as differences in depression, anxiety, and empathy. Recent research indicates that ASD is comorbid with depression in approximately 11% of cases and with anxiety in about 20%, alongside notable empathy deficits (Lai *et al*., 2019; Sharma *et al*., 2018). Thus, scores on STAI, BDI-II, and EQ questionnaires were additionally incorporated as covariates within the GraphVar toolbox, ensuring that the effects of OT on brain connectivity were isolated from these other factors, providing a clearer understanding of how OT interacts with brain networks specifically in association with higher and lower autistic traits.

To explore interaction-dependent changes in brain connectivity, the analysis focused both on individual brain connections and broader network metrics, including global and local measures, as well as mean connectivity strength. At the edge level, NBS were used which are particularly useful for managing family-wise error rates during univariate testing across all connections in the connectivity matrix. This method allowed the identification of subnetworks or topological clusters that showed significant alterations in connectivity between groups, enhancing the sensitivity of the analysis and minimizing false positives. In addition, 1000 permutation tests were performed to assess the robustness of findings, randomly shuffling group labels (higher vs. lower autistic traits) across subjects to confirm that the results were not driven by chance. Sparsity thresholding, ranging from 10% to 50%, was also applied to test the consistency of treatment effects across varying network densities, ensuring that the results were not influenced by a specific threshold but were robust across different network configurations.

To address concerns about multiple comparisons, a false discovery rate (FDR) correction was applied across all thresholds and graph measures. This was critical to control for the potential inflation of Type I errors due to multiple testing, ensuring that the results were statistically valid and reproducible. The significance threshold for all analyses was set at *P* < 0.05, providing confidence that the findings reflected meaningful interaction effects rather than random chance.

For estimating the differences in interaction effects, two-sample two-tailed t-tests were performed on individual graph metrics, including local efficiency, clustering coefficient, degree, nodal path length, and betweenness centrality. The inclusion of comorbidities as covariates in the GraphVar model allowed account to be taken of their influence on FC patterns, ensuring that any observed interaction effects were not confounded by these common variables. This approach helped clarify the true effects of OT on brain connectivity in relation to trait autism, independent of depression, anxiety, and empathy traits.

## Results

### Subject demographics and questionnaire scores

The analysis of the questionnaire data employing two-way analyses of variance (ANOVAs) revealed no significant differences between the higher or lower trait autism groups randomized to the treated with OT or PLC groups on age, psychological traits, mood indices (see Table 1), or head motion (frame displacement). Additionally, higher autistic trait participants across both groups demonstrated minimal levels of depression (mean = 8.19 ± 6.35, range 0–13; Beck *et al*., 1996), mild anxiety levels (mean = 43.80 ± 40.03, scores <55 indicate mild; Liebowitz, 1987), and average empathy levels (mean = 31.36 ± 37.10, range 22–38; Baron-Cohen and Wheelwright, 2004), suggesting that any observed effects were not due to baseline differences in mood or psychological traits (see Table 1).

**Table 1:** Demographics and trait and mood questionnaire scores.

Behavior measures (mean ± SD)	OT higher autistic traits (*n* = 62)	OT lower autistic traits (*n* = 94)	PLC higher autistic traits (*n* = 47)	PLC lower autistic traits (n = 91)	*F*	*P*-value
Age	21.64 ± 2.21	21.77 ± 2.29	22.12 ± 2.79	21.06 ± 2.17	0.52	0.736
EQ score	31.36 ± 11.47	46.38 ± 12.76	37.10 ± 13.29	40.76 ± 13.56	1.08	0.368
ASQ score	28.58 ± 4.09	14.69 ± 2.90	27.12 ± 4.06	14.11 ± 2.78	1.46	0.187
BDI score	8.19 ± 6.31	3.21 ± 3.32	6.38 ± 5.43	5.43 ± 5.57	1.04	0.310
STAI-T score	43.80 ± 9.69	35.63 ± 8.15	40.03 ± 9.85	39.82 ± 9.77	0.57	0.802
PANAS (POS)	24.26 ± 7.48	29.90 ± 7.35	27.07 ± 7.92	27.20 ± 7.93	0.84	0.928
PANAS (NEG)	16.72 ± 6.61	12.87 ± 4.25	15.05 ± 5.79	14. 87 ± 5.83	0.45	0.532

Abbreviations: Becks Depression Inventory (BDI), State-trait Anxiety Inventory (STAI-T), only for trait score, EQ, Empathy Quotient; ASQ, Autism Spectrum Quotient; Positive and Negative Affect Schedule (PANAS), SD, standard deviation; (POS), positive; (NEG), negative; OT, oxytocin; PLC, placebo. *P* values are for treatment × trait autism score interactions.

### Differences in effects of OT on functional connectivity patterns/network edges between individuals with higher and lower autistic traits

**Figure 2:**
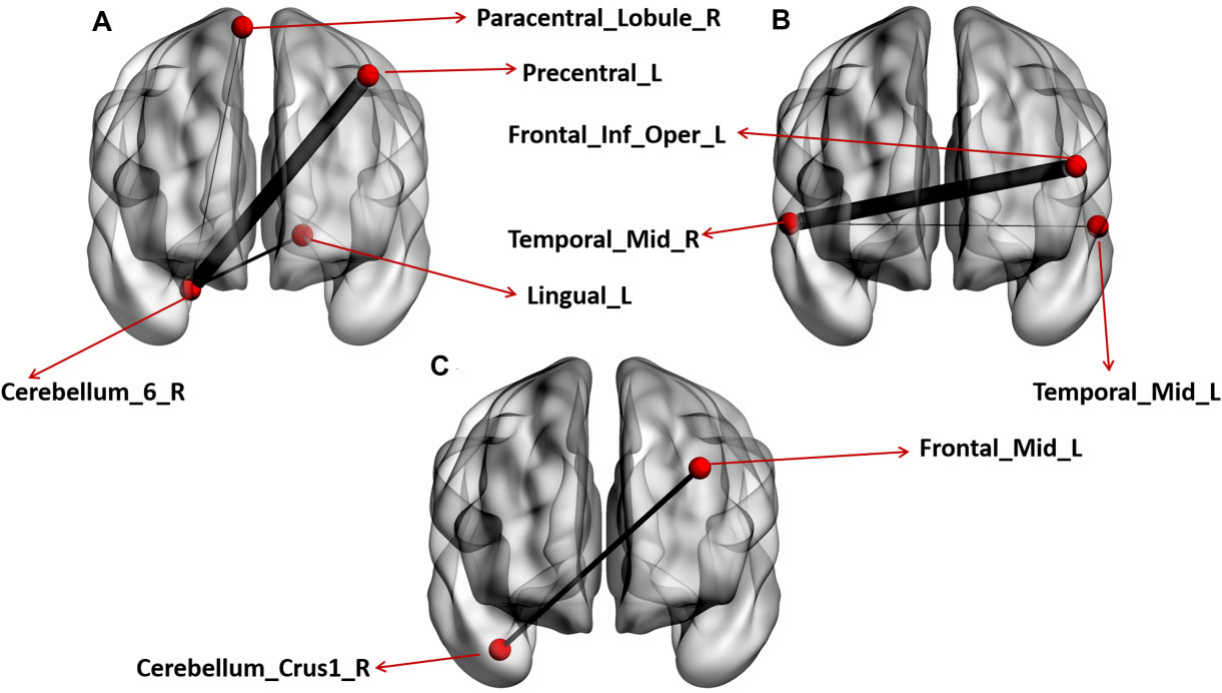
Interaction effect of OT on functional connectivity patterns between higher and lower autistic trait groups. After applying multiple FDR corrections (*P* < 0.05), significant interaction effects on functional patterns were identified. (**A, B, C**) A strengthened interaction effect of OT on functional patterns in individuals with higher autistic traits compared to those with lower autistic traits. Red arrows point to brain regions where OT had a strengthened interaction effect. A first-order interaction model was used within the GraphVar toolbox, which allowed assessment of how the impact of OT on FC differed depending on whether participants had higher or lower autistic traits.

The ANCOVA revealed significant interaction effects on functional connectivity patterns between multiple brain regions after multiple FDR corrections (*P* < 0.05). Specifically, strengthened functional connectivity inter-regional patterns were observed in individuals with higher autistic traits compared to those with lower autistic traits following OT administration. The regions with significantly strengthened functional interaction effects included: Cerebellum_6_R and Precentral_L (*r* = 4.846, *P* = 0.001), Cerebellum_6_R and Lingual_L (*r* = 2.707, *P* = 0.001), Cerebellum_6_R and Paracentral_Lobule_R (*r* = 2.382, *P* = 0.001), Temporal_Mid_R and Frontal_Inf_Oper_L (*r* = 4.536, *P* = 0.001), Temporal_Mid_R and Temporal_Mid_L (*r* = 1.557, *P* = 0.001), and Cerebellum_Crus1_R and Frontal_Mid_L (*r* = 13.649, *P* = 0.001) (see Fig. 2).

### Differences in effects of OT on global and nodal metrics between individuals with higher and lower autistic traits

The ANCOVA focused on the impact of OT on the interaction effects between global and nodal metrics in individuals with higher and lower autistic traits, as well as the differences in these effects across sparsity thresholds ranging from 10% to 50%. After applying multiple FDR corrections (*P* < 0.05), the following significant interaction effects on the global and nodal metric measures were observed (see Tables 2 and 3).

**Table 2:** Interaction effects of OT on the clustering coefficient, local efficiency, and nodal path length in individuals with higher and lower autistic traits.

Threshold	Clustering coefficient	Direction	*R*-value	*P* (FDR-corr)
0.12	Cerebellum_10_R	OT > PL in H; OT < PL in L	8.208	0.001
0.18	Temporal_Inf_R	OT > PL in H; OT < PL in L	3.631	0.001
0.18	Fusiform_R	OT > PL in H; OT < PL in L	2.673	0.001
0.22	Vermis_7	OT > PL in H; OT < PL in L	1.837	0.001
0.26	Pallidum_R	OT > PL in H; OT < PL in L	5.082	0.001
0.29	Rolandic_Oper_R	OT > PL in H; OT < PL in L	3.963	0.001
0.32	Vermis_9	OT > PL in H; OT < PL in L	6.176	0.001
0.33	Cingulum_Ant_R	OT > PL in H; OT < PL in L	6.226	0.001
0.34	Pallidum_L	OT > PL in H; OT < PL in L	5.345	0.001
0.40	Cerebellum_10_L	OT > PL in H; OT < PL in L	3.599	0.001
0.41	Heschl_L	OT > PL in H; OT < PL in L	6.169	0.001
0.44	Cerebellum_4_5_L	OT > PL in H; OT < PL in L	10.175	0.001
0.47	Rolandic_Oper_R	OT > PL in H; OT < PL in L	6.169	0.001
0.48	Fusiform_R	OT > PL in H; OT < PL in L	4.468	0.001
**Threshold**	**Local efficiency**	**Direction**	***R*-value**	***P* (FDR-corr)**
0.11	Right Cuneus	OT > PL in H; OT < PL in L	4.490	0.001
0.12	Right Calcarine	OT < PL in H; OT > PL in L	−31.726	0.001
0.14	Frontal_Sup_L	OT < PL in H; OT > PL in L	−3.765	0.001
0.21	Parahippocampal_R	OT > PL in H; OT < PL in L	5.143	0.001
0.21	Cuneus_L	OT > PL in H; OT < PL in L	7.735	0.001
0.21	Occipital_Inf_R	OT > PL in H; OT < PL in L	4.490	0.001
0.22	Occipital_Inf_L	OT > PL in H; OT < PL in L	5.082	0.001
0.22	Poscentral_L	OT > PL in H; OT < PL in L	3.963	0.001
0.23	Cerebellum_4_5_R	OT > PL in H; OT < PL in L	10.714	0.001
0.25	SupraMarginal_L	OT > PL in H; OT < PL in L	4.247	0.001
0.26	Temporal_Pole_Mid_R	OT > PL in H; OT < PL in L	5.082	0.001
0.31	Frontal_Sup_R	OT > PL in H; OT < PL in L	7.099	0.001
0.31	Vermis_4_5	OT > PL in H; OT < PL in L	4.734	0.001
0.32	Olfactory_R	OT > PL in H; OT < PL in L	4.917	0.001
0.35	Cerebellum_10_R	OT > PL in H; OT < PL in L	7.912	0.001
0.41	Cuneus_L	OT > PL in H; OT < PL in L	5.865	0.001
0.49	Frontal_Med_Orb_L	OT > PL in H; OT < PL in L	5.085	0.001
**Threshold**	**Nodal path length**	**Direction**	***R*-value**	***P* (FDR-corr)**
0.10	Occipital_Mid_R	OT > PL in H; OT < PL in L	5.143	0.001
0.13	Cingulum_Post_R	OT > PL in H; OT < PL in L	5.143	0.001
0.23	Cerebellum_6_R	OT > PL in H; OT < PL in L	6.712	0.001
0.26	Vermis_7	OT > PL in H; OT < PL in L	4.285	0.001
0.29	Temporal_Pole_Sup_R	OT > PL in H; OT < PL in L	5.412	0.001
0.32	Cerebellum_8_L	OT > PL in H; OT < PL in L	5.412	0.001
0.39	Vermis_1_2	OT > PL in H; OT < PL in L	4.154	0.001
0.40	Vermis_9	OT > PL in H; OT < PL in L	7.721	0.001
0.40	Vermis_10	OT > PL in H; OT < PL in L	7.721	0.001
0.47	Vermis_6	OT > PL in H; OT < PL in L	5.412	0.001
0.48	Vermis_4_5	OT > PL in H; OT < PL in L	5.412	0.001
0.48	SupraMarginal_R	OT > PL in H; OT < PL in L	2.919	0.001
0.48	Fusiform_R	OT > PL in H; OT < PL in L	3.050	0.001

FDR-corr indicates FDR-corrected *P* values, all <0.001; OT, oxytocin; PL, placebo; H, higher trait; L, lower trait. For autistic traits, the topological interaction effect of treatment compared the difference values (higher trait minus lower trait) between groups is shown.

**Table 3:** Interaction effects of OT on nodal degree and betweeness centrality in individuals with higher and lower traits.

Threshold	Nodal degree	Direction	*R*-value	*P* (FDR- corr)
0.17	Left Amygdala	OT > PL in H; OT < PL in L	1.389	0.001
0.18	Left Amygdala	OT > PL in H; OT < PL in L	3.300	0.001
0.19	Cerebelum_6_R	OT > PL in H; OT < PL in L	3.300	0.001
0.22	Temporal_Inf_R	OT > PL in H; OT < PL in L	1.913	0.001
0.24	Temporal_Mid_R	OT < PL in H; OT > PL in L	−3.316	0.001
0.25	Cerebellum_Crus2_R	OT < PL in H; OT > PL in L	−3.316	0.001
0.32	Cerebelum_6_R	OT > PL in H; OT < PL in L	2 227	0.001
0.34	Cerebellum_Crus2_R	OT > PL in H; OT < PL in L	3.110	0.001
0.38	Left Amygdala	OT > PL in H; OT < PL in L	4.186	0.001
**Threshold**	**Betweenness centrality**	**Direction**	***R*-value**	***P* (FDR-corr)**
0.11	Cerebellum_Crus1_L	OT > PL in H; OT < PL in L	3.927	0.001
0.13	Cerebellum_Crus2_L	OT < PL in H; OT > PL in L	−7.431	0.001
0.13	Cerebellum_Crus2_R	OT < PL in H; OT > PL in L	−4.787	0.001
0.14	Cerebellum_6_R	OT > PL in H; OT < PL in L	2.892	0.001
0.17	Cerebellum_Crus1_R	OT > PL in H; OT < PL in L	6.023	0.001
0.17	Cerebellum_Crus2_R	OT > PL in H; OT < PL in L	3.535	0.001
0.18	Cerebellum_Crus1_R	OT > PL in H; OT < PL in L	3.865	0.001
0.21	Cerebellum_Crus2_R	OT > PL in H; OT < PL in L	4.329	0.001
0.24	Cerebellum_Crus1_R	OT > PL in H; OT < PL in L	4.177	0.001
0.25	Temporal_Mid_R	OT < PL in H; OT > PL in L	−13.967	0.001
0.26	Rectus_L	OT > PL in H; OT < PL in L	4.504	0.001
0.30	Cerebellum_Crus1_R	OT > PL in H; OT < PL in L	3.103	0.001
0.33	Cerebellum_Crus1_R	OT > PL in H; OT < PL in L	7.267	0.001
0.34	Cerebellum_Crus1_R	OT < PL in H; OT > PL in L	−2.885	0.001
0.34	Cerebellum_Crus1_L	OT > PL in H; OT < PL in L	6.601	0.001
0.35	Occipital_Mid-L	OT > PL in H; OT < PL in L	6.699	0.001
0.41	Temporal_Inf_R	OT > PL in H; OT < PL in L	4.913	0.001
0.42	Paracentral_Lobule-L	OT > PL in H; OT < PL in L	4.998	0.001
0.45	Cerebellum_Crus2_L	OT > PL in H; OT < PL in L	4.563	0.001
0.46	Pallidum_L	OT > PL in H; OT < PL in L	5.912	0.001

FDR-corr indicates FDR-corrected *P* values, all <0.001; OT, oxytocin; PL, placebo; H, higher trait; L, lower trait. For autistic traits, the topological interaction effect of treatment compared the difference values (higher trait minus lower trait) between groups is shown.

### Global efficiency

Interaction effects on the global efficiency measure in individuals with autistic traits administered OT relative to PLC revealed no significant differences in both the higher and lower autistic trait groups.

### Clustering coefficient

**Figure 3:**
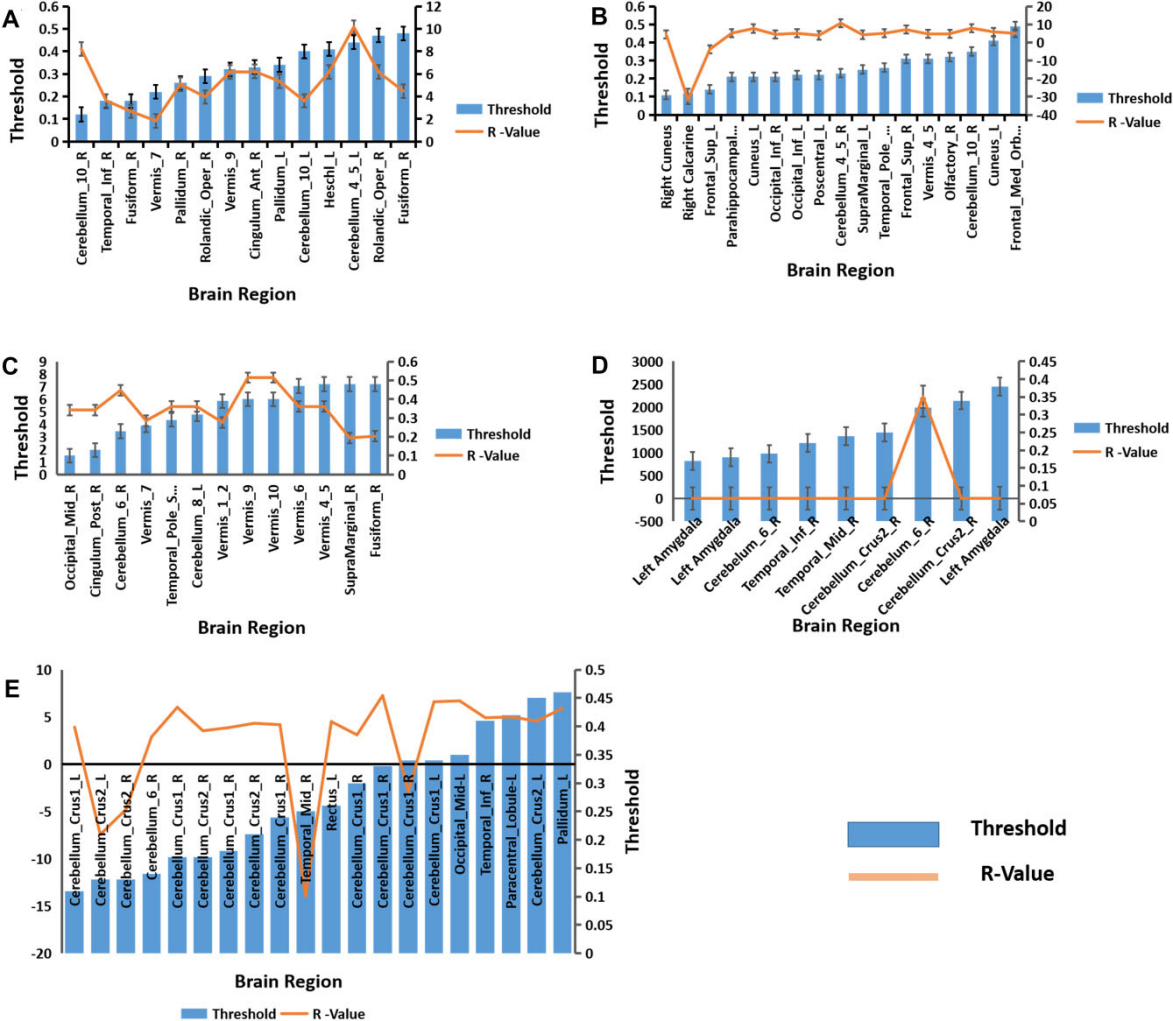
Interaction effect of OT across nodal topology measures in individuals with higher and lower autistic traits. (**A**) Clustering coefficient; (**B**) local efficiency; (**C**) nodal path; (**D**) nodal degree; and (**E**) betweenness centrality. The interaction effect of OT between higher traits compared to lower traits on the global and nodal metrics using a two-sample t-test is shown. After FDR multiple corrections (*P* < 0.05), the displayed brain regions were significant. The blue bars represent sparsity threshold while the orange line represents the *R*-values. All detailed corresponding statistics are presented in Tables 2 and 3.

Significant interaction effects were observed for the local clustering coefficient with OT relative to PLC strengthening it in the Cerebellar Vermis 9, Cerebelum_10_R, Cerebelum_9_R, Temporal_Inf_L, Fusiform_R, Cerebellar Vermis 7, Pallidum_R, Pallidum_L, Cingulum_Ant_R, Rolandic_Oper_R, Cerebelum_10_L, and Heschl_L regions in individuals with higher autistic traits compared to those with lower ones at various sparsity levels (see Table 2 and Fig. 3A).

### Local efficiency

Significant interaction effects in the local efficiency of brain regions were observed with OT relative to PLC, demonstrating strengthening local efficiency in the Cuneus_R, Parahippocampal_R, Cuneus_L, Occipital_Inf_R, Occipital_Inf_L, Poscentral_L, Cerebellum_4_5_R, Supramarginal-L, Temporal_Pole_Mid_R, Frontal_Sup_R, Vermis_4_5, Olfactory_R, Cerebellum_10_R, Cuneus_L, and Frontal_Med_Orb_L but weakened it in Calcarine_R and Frontal_sup_L regions in higher autistic trait individuals compared to lower trait ones across various sparsity levels (see Table 2 and Fig. 3B).

### Nodal path length

Significant interaction effects in the nodal path length of brain regions were observed in higher autistic trait compared to lower autistic trait individuals. Specifically, relative to PLC, OT administration led to greater nodal path length shortening in the Occipital_Mid_R, Cingulum_Post_R, Cerebelum_6_R, Cerebellar Vermis 7, Temporal_Pole_Sup_R, Cerebelum_8_L, Vermis_1_2, Vermis_9, Vermis_10, Vermis_6, Vermis_4_5, SupraMarginal_R, and Fusiform_R across various sparsity levels (see Table 2 and Fig. 3C).

### Nodal degree

Significant interaction effects in nodal degree were observed in higher autistic trait compared to lower autistic trait individuals. Specifically, OT relative to PLC increased nodal degree in the left amygdala, Cerebellum_6_R, and Temporal_Inf_R but reduced it in Cerebelum_Crus2_R and Temporal_Mid_R across various sparsity levels in individuals with higher autistic traits relative to those with lower autistic ones (see Table 3 and Fig. 3D).

### Betweenness centrality

Significant interaction effects were observed in betweeness centrality in OT relative to PLC with either strengthening occurring in the Cerebellum_Crus1_L and _R, Cerebellum_6_R, Rectus_L, Occipital_Mid_L, Temporal_Inf_R, Pallidum_L, and Paracentral_Lobule_L or weakening in Cerebellum_Crus2_L and _R and Temporal_Mid_R regions across various sparsity levels in higher trait individuals compared to lower trait ones (see Table 3 Fig. 3E).

## Discussion

The current study employed an exploratory, data-driven approach to investigate the effects of intranasal OT on FC patterns and network topology organization between individuals with higher compared to lower autistic traits. The findings on FC interactions revealed significantly strengthened and weakened connectivity patterns within the resting-state sensorimotor, attention, cognitive, emotion, and language processing networks. Moreover, interaction effects on network topology revealed significant alterations in local but not global graph metrics in individuals with higher autistic traits compared to those with lower traits, across multiple brain regions. These regions included the left amygdala, right parahippocampal gyrus, cerebellum, left calcarine, cerebellar, and fusiform gyrus regions, all of which are localized to the DMN, social cognition, sensorimotor, cognitive, and emotion networks and are highly implicated in the pathophysiology of ASD (Ha *et al*., 2015; Itahashi *et al*., 2014). Overall, the findings align with prior meta-analytic studies on the impact of OT on social brain networks (Schurz *et al*., 2021) and recent studies indicating that OT modulates the intrinsic dynamics of large-scale brain networks involved in social, emotional, and attentional processing, critical areas often disrupted in ASD (Jiang *et al*., 2021; Xin *et al*., 2021).

In terms of the effects of OT on FC, a number of connections were strengthened but only in individuals with higher trait autism. Pathways with strengthened FC particularly involved those between cerebellar regions and regions involved in sensorimotor function (precentral gyus, lingual gyrus, and parabrachial lobule), emotional cue, and language processing (left inferior frontal operculum). Interestingly, several previous resting state studies have reported effects of OT on cerebellum FC with one study reporting decreased connectivity with reward areas (putamen) (Zhao *et al*., 2019), and another increased connectivity with the amygdala (Eckstein *et al*., 2017) which might suggest differential effects on reward and emotional control. FC between the middle temporal gyrus and inferior frontal operculum and between the left and right mid-temporal cortices was also strengthened and these pathways are important for attention, emotional regulation, and cognitive and language processing (Diveica *et al*., 2021; Friederici, 2011; Wang *et al*., 2024). These FC findings suggest that OT may enhance the integration of sensory, visual, cognitive, and emotional control, language, and motor functions, domains frequently disrupted in individuals with higher autistic traits (Paul *et al*., 2021) or clinical ASD (Baum *et al*., 2015; Bhat, 2021; Ha *et al*., 2015). This is in broad agreement with our previous study which did not take into account individual autistic traits (Hagan *et al*., 2025), although in this previous study OT was observed to weaken FC in a broader range of pathways possible reflecting it having opposite effects on FC in individuals with lower as opposed to higher autistic traits. The findings also align with studies demonstrating that OT could improve sensorimotor functions in ASD, potentially enhancing both social engagement and motor coordination (Amonkar *et al*., 2021) and improvements in cognitive flexibility following OT administration in individuals with ASD (Han *et al*., 2023).

Notably, as in our previous study (Hagan *et al*., 2025), we did not find evidence for OT strengthening FC between medial prefrontal areas and the amygdala, important for emotional control, in contrast to several previous studies (Ebner *et al*., 2016; Kou *et al*., 2022; Sripada *et al*., 2013). However, most of these latter studies adopted a region of interest rather than the whole-brain approach used in the current study.

### Differences in the effects of OT on global and local network topology properties between individuals with higher and lower autistic traits

Advanced graph theoretical methods were used to assess global and local network topology metrics in terms of nodes and edges. No significant effects of OT were observed for global topology network measures for either higher or lower trait autism individuals in line with our previous study (Hagan *et al*., 2025). On the other hand, the current study revealed that OT primarily enhances local network processing properties, improving metrics such nodal path length and degree, local efficiency, clustering coefficients, and betweeness centrality across sparsity thresholds, indicating that it improves processing efficiency and integration in local brain networks (Park and Friston, 2013). In line with our previous study, which did not take into account trait autism scores (Hagan *et al*., 2025), the most notable regions showing improvements in all of these local processing measures were multiple regions of the cerebellum and vermis and the occipital cortex, suggesting extensive modulation within motor and visual processing systems. The most widespread changes were in local efficiency, a measure of the efficiency of information transfer in each node's neighborhood, of networks not only in motor (post-central gyrus as well as cerebellum) and visual systems (cuneus as well as occipital cortex) but also in the DMN (medial and superior frontal cortices and parahippocampal gyrus) and socio-emotional processing regions (temporal pole, supramarginal gyrus). Local clustering coefficients, which quantify how close the neighbors of a node are to being a complete graph, were again strengthened in motor and visual processing regions but also in auditory processing and language (Heschl's gyrus, Rolandic operculum), visual social recognition (inferior temporal and fusiform cortices), and emotional (cingulum) and sub-cortical reward processing (pallidum) regions. These regions and functions are often altered in ASD (Bhat, 2021; Ha *et al*., 2015) and thus OT may enhance local integration of these networks not only in individuals with higher autistic traits but also potentially in individuals with clinical ASD, who typically face greater challenges in these domains. This finding is in line with previous studies, showing that OT can positively modulate brain structural and functional connectivity in regions associated with emotion regulation, social cognition, and reward in individuals with ASD (Alaerts *et al*., 2019, 2024; Gordon *et al*., 2013, 2016). Additionally, the influence of OT on regions such as the left pallidum and posterior cingulate highlights its potential to facilitate local processing in areas critical for emotional awareness and regulation and reward, which are often disrupted in ASD (Baum *et al*., 2015; Mazefsky *et al*., 2013; Scott-Van Zeeland *et al*., 2010). These network-specific effects of OT modulation offer a foundation for understanding its potential clinical relevance.

Clinically, the implications of these findings are substantial, as they reveal that the effects of OT on brain connectivity occur particularly in neurotypical individuals with higher autistic traits and this strongly suggests that OT could have similar effects in individuals diagnosed with clinical autism. Our data indicate that OT administration selectively enhances local neural network integration within brain regions central to social cognition, emotional regulation, and sensorimotor processing domains commonly disrupted in autism. This localized modulation may confer clinically meaningful improvements such as increased social engagement, better emotional self-regulation, and improved motor coordination. These findings offer a neurobiological rationale for targeting OT-based interventions to individuals who demonstrate specific neural connectivity profiles or trait dimensions associated with autism.

Notably, we identified neural markers—such as increased local efficiency and clustering in regions such as the cerebellum, fusiform gyrus, and amygdala—that responded to OT in individuals with higher autistic traits. These markers have potential utility in clinical practice since they could serve as predictive indicators of treatment response or as targets for monitoring therapeutic outcomes. It is important to acknowledge, however, that this study employed a dimensional, trait-based approach within a neurotypical sample, rather than a clinically diagnosed ASD population. While this aligns with the RDoC (Research Domain Criteria) framework and enhances our understanding of the full spectrum of autistic traits, it does still require a further translational step. Encouragingly, converging evidence from prior studies has demonstrated similar OT-related neural effects in both subclinical and clinical ASD populations (e.g. Gordon *et al*., 2016; Alaerts *et al*., 2019), suggesting the generalizability of our findings. Nevertheless, direct comparisons involving clinically diagnosed individuals using identical protocols are essential for validating and extending these results in therapeutic contexts.

Nevertheless, the effects of OT were not uniform across all brain regions or individuals in the local efficiency metric. For instance, the visual network, represented by the right calcarine region, exhibited weakened interaction effects in individuals with higher autistic traits, suggesting that the impact of OT on sensory integration may be limited in those with pronounced sensory processing difficulties, a hallmark of ASD (Chen *et al*., 2024; Marco *et al*., 2011). In contrast, OT enhanced local network efficiency in other visual regions such as the right cuneus as well as regions in the DMN involved in memory processing, the right parahippocampal gyrus, and the cerebellum involved in motor control as well as cognitive and emotion processing functions.

Interestingly, the betweenness centrality metric further demonstrated the complexity of the impact of OT. Betweenness centrality represents the degree to which nodes stand between each other with increased centrality of a node indicating that it can exert more control over the network because more information will pass through it. Strengthened interaction effects were observed in primary visual (left middle occipital cortex) and visual association regions involved in social recognition (right inferior temporal cortex), indicating that OT may improve sensory processing by enhancing integration between these regions. These results align with prior studies suggesting that OT can facilitate sensory integration, particularly in individuals with ASD (Korisky *et al*., 2022). Conversely, weakened interactions in the social cognition network (middle temporal gyrus) underscore the complexity of the influence of OT on higher-order processes, such as social cognition and language, and highlights the need for further exploration of its region-specific effects in relation to autism.

The nodal path length (how long the connection is between adjacent nodes) and nodal degree (the number of connections to other nodes in the network) metrics also revealed effects of OT in higher trait autism individuals. Specifically, for nodal length, OT shortened path lengths in primary visual (medial occipital cortex) and visual association areas involved in social recognition (fusiform gyrus) as well as in the cerebellum and vermis, posterior cingulate, and supramarginal gyrus involved in motor, cognitive, and emotional control. For nodal degree, increases were found in the left amygdala, a key region within the limbic network involved in emotional processing and regulation, again suggesting that OT may enhance emotional processing, consistent with findings that it improves social cognition and emotional regulation (Yao and Kendrick, 2025). This could reflect the potential of OT to modulate hyperactivity and improve socio-emotional functioning, as supported by previous research on the effects of OT on amygdala connectivity and responses to emotional stimuli (Jiang *et al*., 2021; Kou *et al*., 2022). However, interestingly, nodal degree was also reduced in the cerebellum and middle temporal cortex, again suggesting that in some instances OT may be reducing the number of connections in specific networks.

The absence of any significant effects of OT on global topology measures indicate that it may not be that effective in influencing global processing dysfunction in the brain which has been reported by some studies in individuals with ASD (Keown *et al*., 2017; Rudie *et al*., 2012). Analysis of dynamic changes in local and global processing have revealed differences between ASD and neurotypical individuals (Talesh Jafadideh and Mohammadzadeh Asl, 2022) and it is possible that a similar dynamic analysis of OT effects on topological measures might reveal modulation of global processing. Additionally, the current study only investigated the effects of a single acute dose of intranasal OT whereas when it is used in a therapeutic context it is administered repeatedly over many weeks. Possibly, chronic OT treatment might therefore influence global as well as local processing in neural networks.

The current study has some limitations. First, only male participants were included and given evidence that OT can have different effects in males and females (Yao and Kendrick, 2025), further studies will need to establish the possibility of sex differences in its effects on FC and local network topology. Second, the study used a dimensional approach to establish that OT in particular influences FC and network topology changes in individuals with higher trait autism but these findings will need to be confirmed in individuals diagnosed with clinical ASD. Third, only the effects of a single dose of OT were analyzed and it will be important to investigate whether chronic dosing protocols typically used in therapeutic contexts might have different actions on neural processing.

## Conclusion

This study provides robust evidence that intranasal OT administration significantly modulates FC and network topology in individuals with higher but not lower autistic traits. The observed changes in brain regions associated with social cognition, emotion regulation, and sensorimotor integration underscore the therapeutic potential of OT for addressing social and emotional problems in ASD. These findings advance our understanding of the neurobiological mechanisms underlying social behavior in autism and highlight the potential of OT as a targeted intervention. Future studies should aim to replicate and extend these findings in larger, more diverse cohorts, exploring the long-term effects of OT administration on functional brain networks and behavioral outcomes in a clinical sample. By elucidating the neural mechanisms underlying the therapeutic effects of OT, this research has the potential to inform personalized treatment approaches, ultimately improving the quality of life for individuals with ASD.
